# Trastuzumab anti-tumor efficacy in patient-derived esophageal squamous cell carcinoma xenograft (PDECX) mouse models

**DOI:** 10.1186/1479-5876-10-180

**Published:** 2012-08-30

**Authors:** Xianhua Wu, Jingchuan Zhang, Ruheng Zhen, Jing Lv, Li Zheng, Xinying Su, Guanshan Zhu, Paul R Gavine, Songtao Xu, Shaohua Lu, Jun Hou, Yalan Liu, Chen Xu, Yunshan Tan, Liang Xie, Xiaolu Yin, Deming He, Qunsheng Ji, Yingyong Hou, Di Ge

**Affiliations:** 1 Department of Thorax Surgery, Zhongshan Hospital, Fudan University, Shanghai 200032, P. R. China; 2 Innovation Center China, AstraZeneca Global R&D, Zhangjiang Hi-Tech Park, Shanghai 201203, P. R. China; 3 Department of Pathology, Zhongshan Hospital, Fudan University, Shanghai 200032, P. R. China

**Keywords:** Esophageal carcinoma, HER-2, Herceptin, *PIK3CA* mutation, Xenograft model

## Abstract

**Background:**

Trastuzumab is currently approved for the clinical treatment of breast and gastric cancer patients with HER-2 positive tumors, but not yet for the treatment of esophageal carcinoma patients, whose tumors typically show 5 ~ 35% HER-2 gene amplification and 0 ~ 56% HER-2 protein expression. This study aimed to investigate the therapeutic efficacy of Trastuzumab in patient-derived esophageal squamous cell carcinoma xenograft (PDECX) mouse models.

**Methods:**

PDECX models were established by implanting patient esophageal squamous cell carcinoma (ESCC) tissues into immunodeficient (SCID/nude) mice. HER-2 gene copy number (GCN) and protein expression were determined in xenograft tissues and corresponding patient EC samples by FISH and IHC analysis. Trastuzumab anti-tumor efficacy was evaluated within these PDECX models (n = 8 animals/group). Furthermore, hotspot mutations of EGFR, K-ras, B-raf and PIK3CA genes were screened for in the PDECX models and their corresponding patient’s ESCC tissues. Similarity between the PDECX models and their corresponding patient’s ESCC tissue was confirmed by histology, morphology, HER-2 GCN and mutation.

**Results:**

None of the PDECX models (or their corresponding patient’s ESCC tissues) harbored *HER-2* gene amplification. IHC staining showed HER-2 positivity (IHC 2+) in 2 PDECX models and negativity in 3 PDECX models. Significant tumor regression was observed in the Trastuzumab-treated EC044 HER-2 positive model (IHC 2+). A second HER-2 positive (IHC 2+) model, EC039, harbored a known PIK3CA mutation and showed strong activation of the AKT signaling pathway and was insensitive to Trastuzumab treatment, but could be resensitised using a combination of Trastuzumab and AKT inhibitor AZD5363. In summary, we established 5 PDECX mouse models and demonstrated tumor regression in response to Trastuzumab treatment in a HER-2 IHC 2+ model, but resistance in a HER-2 IHC 2+/PIK3CA mutated model.

**Conclusions:**

This study demonstrates Trastuzumab-induced tumor regressions in HER-2 positive tumors, and highlights PIK3CA mutation as a potential resistance mechanism to Trastuzumab treatment in pre-clinical patient-derived EC xenograft models.

## Introduction

The incidence of esophageal carcinoma (EC) and the histological type varies widely with geographical location [1]. Notably, esophageal squamous cell carcinoma (ESCC) is the predominant histological type in Asian areas with a high risk of cancer development in the esophagus, in contrast to Western countries (Northern Europeans and Caucasians in the USA) [1]. In the United States and North and Western Europe, where esophageal adenocarcinoma is the predominant histological type, the incidence of ESCC has been decreasing in recent decades, whilst the incidence of esophageal adenocarcinomas, especially of the gastroesophageal junction, has been increasing [2,3].

Surgery remains the mainstay of therapy for esophageal carcinoma patients. However, ESCC diagnosis is associated with a high mortality owing to its aggressive behavior and the 5-year survival for non-metastatic disease ranges from 20% to 40% [2]. Patients with metastatic disease who are treated with palliative chemotherapy using a combination of 5-Fluorouracil (5-FU) and Cisplatin have a median survival of less than one year and response rates of 25% to 45% in phase II and III trials [4,5]. Salvage options for patients with refractory EC are very limited [6]. Unfortunately, there are no clinically approved targeted therapies for the treatment of esophageal carcinoma.

Human epidermal growth factor receptor 2 (HER-2) is overexpressed in breast and other types of human cancers and has been successfully developed as a therapeutic target [6,7]. Antibody-based therapy with Herceptin (Trastuzumab) has been used for the clinical treatment of HER-2-positive breast cancer [6,7]. Recently, Trastuzumab therapeutic efficacy has also been observed in HER-2-overexpressing gastric cancer [7].

Previous studies in patients with ESCC indicated that the frequency of HER-2 protein expression ranged from 0 to 56% [8,9], whilst the frequency of HER-2 gene amplification ranged from 5 to 35% [8,9]. These data suggested that a proportion of ESCC patients could be candidates for Trastuzumab targeted therapy. Results from early clinical trials demonstrated a correlation between HER-2 expression and gene amplification with Trastuzumab therapeutic efficacy in patients with esophageal adenocarcinomas [10]. However, over expression and amplification of HER-2 appeared to be fundamentally different within esophageal squamous cell carcinoma, with a tendency of lower positive rates and lower level amplification, compared to adenocarcinoma [11]. Accordingly, it is not possible to imply Trastuzumab efficacy within esophageal squamous cell carcinoma, and as such, these studies aimed to address this preclinically. Therefore, we established xenograft mouse models derived from patient’s ESCC tissues and used these novel, clinically relevant ESCC models to explore the anti-tumor efficacy of Trastuzumab targeted therapy.

## Materials and methods

### Patients and tissue samples

ESCC tissues from 54 treatment-naïve patients were obtained intraoperatively during esophagetomy resection at Zhongshan Hospital (Shanghai, China) from March 2010 to June 2010. ESCC histology was confirmed by pathological analysis. Prior written informed consent was obtained from all patients and the study protocol was approved by the ethics committee at Zhongshan hospital, Fudan University. Freshly harvested ESCC specimen was separated into three parts. The first part was placed into medium containing antibiotics immediately after surgical resection under sterile conditions and transported to the animal facility within two hours for implantation into immunodeficient mice. The second part of the specimen was snap frozen immediately in liquid nitrogen for DNA/RNA extraction. The third part of the specimen was fixed in formalin and embedded into paraffin (FFPE) for pathological and IHC analysis.

### Development of patient-derived esophageal carcinoma xenograft (PDECX) mouse models

Female nude (*nu/nu*) (8-10-week-old) and severe combined immune deficient (SCID) mice (Vital River, Beijing, China) were used for xenograft model generation. All experiments with immunodeficient mice were performed in accordance with the guidelines approved by IACUC. The PDECX mouse models were established with fresh ESCC tissues surgically removed from EC patients. Briefly, surgically removed patient’s ESCC tissues (F0 tissue) were cut into fragments of approximately 15 mm^3^ and implanted via Trocar needle subcutaneously into female SCID mice within two hours after the surgery. The implanted mice were observed daily until 90 days. Tumors were measured once a week by caliper to determine its subcutaneous growth. The xenografted ESCC tumors (~500 mm^3^) were harvested from the patient ESCC-bearing mice and were further implanted in female nude mice for expansion. After three consecutive mouse-to-mouse passages, the xenograft was considered to be stabilized and was submitted to the process of model characterization, including histopathologic analysis, HER-2 expression by FISH and IHC assays, and mutation detection for EGFR, K-ras, B-raf and PIK3CA genes. The xenograft tumor specimens in each passage of tumor-bearing mice were harvested and divided into three parts as same as the patient tumor samples. Fresh tumor fragments at passage 3–5 were frozen with 20% FCS in liquid nitrogen for further model recovery. The PDECX models were maintained in nude mice and were used for anti-tumor efficacy studies.

### H&E and immunohistochemical staining

Tissues from all PDECX mouse models and their corresponding ESCC patients were harvested and fixed in 10% buffered formalin within 30 minutes after resection. Tissues were processed following the routine procedure after 24 hours fixation. Sections were stained with Hematoxylin and eosin and reviewed by a pathologist to confirm the ESCC diagnosis. Dual cores from each case were made on tissue microarray (TMA) for IHC and FISH analysis. For the IHC, all incubations were at room temperature and washes were performed with TBST. Tissues sectioned at 4 μm onto slides were dewaxed and rehydrated. Antigen retrieval was performed in a pressure cooker at 110°C for 5 minutes in retrieval buffer (S2367, DAKO). Endogenous peroxidase activity was blocked with 3% hydrogen peroxide (S2023, DAKO). Sections were then incubated with anti-HER-2 antibody (HerceptestTM, DAKO) for 30 minutes. Immunocomplexes were detected by incubation with DAKO K5204 for 30 minutes and were visualized with diaminobenzidine (K3468, DAKO) for 10 minutes. Normal IgG from the same species of primary antibody diluted to match the concentration of the primary antibody was used as the negative control. For the HER-2 negative cases, the experiment was repeated on the whole section in order to exclude heterogeneity.

In the present study, the IHC scoring on EC followed Hoffman’s criteria on human gastric cancer [12]: no staining or <10% tumor cell positive staining were considered as 0/negative; faintly or barely perceptible staining on >10% tumor cell membrane were considered as 1+; weak to moderate positive staining on >10% tumor cells were considered as 2+; since tumor tissue on TMA mimicked the biopsy samples, cohesive moderate to strong staining on the membrane was scored as 3 + .

### HER-2 FISH assay

HER-2/CEP17 probes for Bysis (Cat #30-161060) was used and FISH assay was performed according to the manufacturer’s instructions. In brief, TMA sections were dewaxed and dehydrated. Tissue samples were pretreated following the manual of SpotLight Tissue pretreatment kit (Invitrogen, Carlsbad, CA) (Cat #00-8401). After pretreatment, 10 μl HER-2/CEP17 probes were applied onto each TMA slide, which was subsequently covered with a coverslip and circled with rubber cement (Mpbio, Solon, OH) (Cat #11FIXO0125) around the edge of coverslip. Sections together with probes were codenaturated at 75°C for 4 minutes on OmniSlide In-Situ hybridization System, followed by incubation at 37°C overnight. Probes were washed sequentially with 0.3%NP40/1 × SSC at 75.5°C for 3 min, 0.3%NP40/2 × SSC at 75.5°C for 2 minutes and 2 × SSC at room temperature for 2 min, with twice for each wash. The slides were then dehydrated and mounted with 0.3 μg/ml DAPI mounting medium (Vector, Burlingame, CA) (Cat #H-1200). In each case, 100 tumor nuclei were evaluated based on Hoffmann criteria [12] and other investigator [9]. An absolute HER-2 gene copy number lower than four or an HER-2/Chr17 ratio of less than 1.8 was considered as negative for HER2 gene amplification. Cases with average HER-2 gene copy number >6 or a HER-2/CEN17 fluorescence ratio > or = 2 were considered as positive for gene amplification. Cases with HER-2 copy number between four and six or an HER-2/Chr17 ratio between 1.8 and 2.0 were considered as HER-2 equivocal.

### Anti-tumor efficacy of Trastuzumab treatment

Tumor growth curves were generated by kinetic measurement of tumor volumes. For therapeutic experiments, tumor-bearing mice with a tumor range of 100 to 200 mm^3^ were randomly divided into the vehicle control or Trastuzumab treatment groups (8 animals per group). The mice in the treatment group were treated with monotherapy of Trastuzumab (Roche, China, 4 mg/kg/twice a week intraperitoneally) or combination of Trastuzumab with AKT inhibitor AZD5363 (120 mg/kg/twice daily, orally). Subcutaneous tumors in nude mice and mice body weight were measured twice a week. Tumor volumes were calculated by measuring two perpendicular diameters with calipers. Tumor volumes (V) were calculated by the formula: V = (length + [width]^2^)/2. Percentage of tumor growth inhibition (%TGI = 1 – [changes of tumor volume in treatment group/ changes of tumor volume in control group] × 100) was used for the evaluation of anti-tumor efficacy. For tumor regression, in which the tumor volume after treatment was smaller than the tumor volume at the beginning, the following equation was used: regression% = 100 × (T0-Ti)/T0, Ti and T0 was the tumor volume in the same group but measured at the different time points. T0 is the tumor volume on the day before first dosing of the compound, and Ti is the tumor volume at the last measurement day after compound treatment.

### Screening of gene mutations

Formalin-fixed paraffin-embedded tumor blocks were reviewed for quality and tumor content. A single representative block, from PDECX mouse models and the corresponding patient ESCC samples, containing at least 70% of neoplastic cells, was analysed for gene mutations. Genomic DNA was extracted using the QIAamp Mini kit (Qiagen, Valencia, CA) according to the manufacturer’s instructions. Hotspots in exon 18, 19, 20, 21 of EGFR gene, exon 2 and 3 of K-ras gene, exon 11 and 15 of B-raf gene, and exon 9 and 20 of PIK3CA gene were screened by scorpions amplification refractory mutation system (ARMS) and mutant-enriched liquid chip PCR method [13]. The former method and detection was supported by Amoy diagnostics Co. Ltd, Fujian, PR China, the latter method was completed by SurExam Bio-Tech Co. Ltd., Guangzhou Technology Innovation Base (Science City, Guangzhou, PR China). Positive results in both methods were further confirmed by PCR-direct sequencing. The primers for exon 9 of PIK3CA gene were GTCTTAGATTGGTTCTTTCCTG for forward and GCATTTAATGTGCCAACTACC for reverse, for exon 20 of PIK3CA gene were TTTGTCTACGAAAGCCTCTCTA for forward and CCATCACTTTTTCCTTCTCCAT for reverse.

### Western blot (Wb)

Total proteins were extracted with mammalian protein extraction reagent (Beyotime, P0015, China). 80 μg of total protein was electrophoresed in 4%-12% Criterion XT Bis-Tris gels (Bio-Rad) and transferred to polyvinyl difluoride (PVDF) membranes (Millipore) at 340 mA for 75 min. Membranes were blocked with 5% non-fat milk in TBST buffer and probed with specific antibodies. All primary antibodies were used at a final concentration of 1 μg/ml. The blots were then visualized with a chemiluminescent detection system as described by the manufacturer. Antibodies against HER-2 (#06-562) was purchased from Upstate Biotechnology; p-Mtor-Ser 2448 (No. 2971) from DAKO Biotechnology, and mTor (No 2972), AKT (No 9272), p44/42 MAP Kinase (No 9102), S6 (No 2317), 4EBP1 (No 9452) and p-AKT-Ser 473 (M3628), Phospho-p44/42-MAPK-Thr202/Tyr 204 (No 4370), p-S6-Ser 240/244 (No 2215), p-4EBP1-Thr 70 (No 9455), and anti-GAPDH were purchased from CST Biotechnology.

### Statistical analysis

Statistical significance was evaluated using a one-tailed, two sample *t* test. P values of less than 0.05 were considered to be significant.

## Results

### Establishment of PDECX mouse models

Of the 54 patient ESCC tissues transplanted subcutaneously into immunodeficient mice, a total of 25 (46.3%) PDECX models were successfully established. After screening of HER-2 expression and amplification by IHC and FISH in all 25 models, only EC039 and EC044 showed IHC 2+ for HER-2 expression. HER-2 amplification was not identified in any of the models. EC039, EC044 and 3 HER-2 negative models were selected for the next experiments.

**Figure 1:**
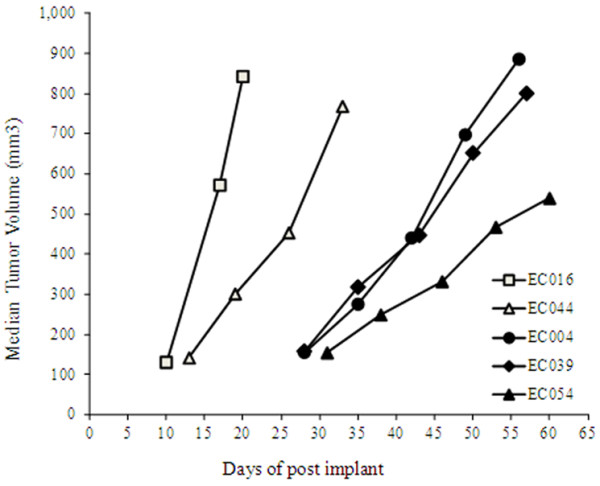
**Tumor growth curves of the 5 PDECX mouse models at passage 3. **Subcutaneous tumors were measured by caliper, and median tumor volumes in 3 tumor-bearing mice from each model are represented. X-axis represents days after tumor implantation.

The features of the 5 ESCC patient models selected for Trastuzumab anti-tumor efficacy testing are shown in Table 1. Prior to surgery, all patients were treatment-naïve. Patient ESCC samples grew up in SCID mice after subcutaneous implantation with the original patient ESCC tissue and continued to grow up in nude mice after a second generation with implantation of xenograft tumor. At the time of study, these PDECX mouse models had more than 5 passages and showed different kinetic growth curves. The latency from implantation of ESCC tissues to the initiation of growth in mice became shorter with increasing passage: 61 ± 22 days in passage 1; 56 ± 28 days in passage 2; 49 ± 29 days in passage 3, and became stable after passage 3. Figure 1 shows the growth curves of the 5 established PDECX mouse models at passage 3. The 5 established PDECX mouse models could be divided into two groups based on latency and *in vivo* tumor doubling time. Two models had short latency periods (24 days in EC016 model, and 35 days in EC044 model), but different tumor doubling times (96 hours in EC016 model, and 192 hours in EC044 model). The other 3 models had longer latency periods (74 ± 23 days) and tumor doubling times (312 ±62 hours). Autopsy examination of tumor-bearing mice from these PDECX models at 2–3 months post-implantation revealed no evidence of metastases to the stomach, brain, lung, liver or kidney. All 5 PDECX mouse models and their corresponding patient’s EC tissues were pathologically confirmed to be squamous cell carcinoma. Similar histological features were observed between each xenograft model and the corresponding patient’s ESCC tissues (data not shown).

**Table 1:** Patient and corresponding PDECX mouse model information

**Patient information**					**Characterization & anti-tumor efficacy in the Patient-derived EC xenograft models**									
					**HER-2**			**Mutation**				**Trastuzumab efficacy**		
**Gender/age**	**Pathology**	**TNM stage**	**Grade**	**Model ID**	**FISH GCN**	**FISH Ratio (HER-2/CEP17)**	**IHC**	**PIK3CA**	**EGFR**	**K-ras**	**B-raf**	**TGI%**	**Tumor regression %**	**P value**
M/70	ESCC	IIIB	Moderate	EC004	1.52	1.07	-	-	-	-	-	44	0	0.1333
M/61	ESCC	IIA	Poor	EC016	2.38	1.25	-	-	-	-	-	0	0	0.3113
M/65	ESCC	IIA	Moderate	EC039	3.92	1.26	++	+	-	-	-	41	0	0.2290
M/56	ESCC	IIIA	Moderate	EC044	3.90	1.55	++	-	-	-	-	>100	93	<0.0001
M/73	ESCC	IIIA	Moderate	EC054	3.04	1.27	-	-	-	-	-	48	0	0.0004

### Characterization of HER-2 expression and gene copy number in PDECX models

**Figure 2:**
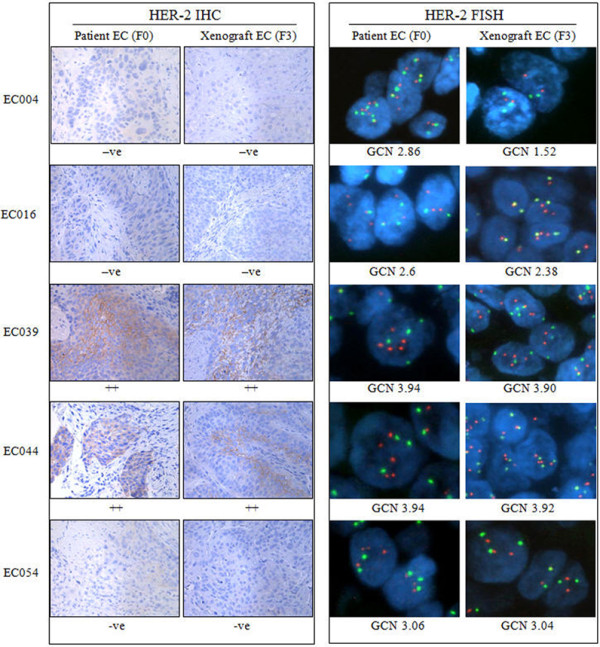
**Representative pictures of HER-2 IHC and FISH staining on models EC004, 016, 039, 044 and 054 with matched human primary tumor tissues. **HER-2 moderate staining (++) and FISH GCN increase were detected in models EC039 and EC044 and matched primary tumor tissues. EC004, EC016 and EC054 were shown to be HER-2 IHC negative and FISH non-AMP.

Following Hoffman’ HER-2 score criteria (12), two PDECX mouse models (EC039 and EC044) were scored as moderately positive for HER-2 membrane staining (2+), the remaining models (EC004, 016 and 054) were negative for HER-2 staining (Figure 2). According to Hoffman’s criteria, none of the PDECX models were identified as HER-2 gene amplified using FISH analysis. However, models EC039 and EC044 showed increased gene copy numbers, with average copy numbers of 3.90 and 3.92, respectively. HER-2 expression and gene copy number were similar between the PDECX models and their corresponding patient’s ESCC tissues (Figure 2).

### Trastuzumab antitumor efficacy in PDECX models

**Figure 3:**
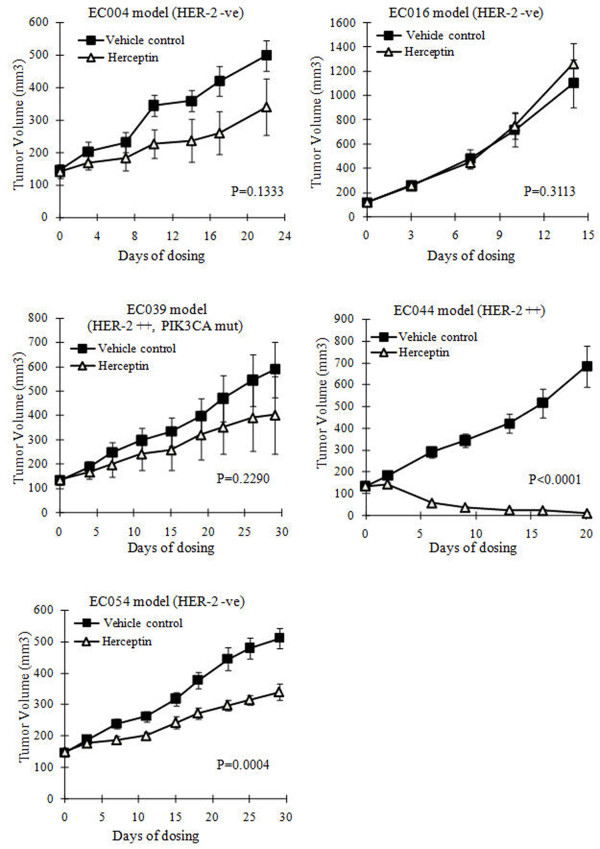
**Anti-tumor efficacy of Trastuzumab in the 5 PDECX mouse models. **Tumor-bearing mice were treated starting from tumor volume 100 ~ 200 mm^3^. The subcutaneous tumor volume was measured by caliper and calculated as mean ± SEM. Y-axis represented the volume of the tumor (mean ± SEM), and X-axis represents the number of days following treatment start day.

Trastuzumab anti-tumor efficacy was assessed in all 5 PDECX mouse models (Figure 3 and Table 1). Three HER-2 negative PDECX mouse models (EC004, 016 and 054) showed either no or marginal responses to Trastuzumab treatment (ranging from 0 to 48% TGI). Only one HER-2 negative model (EC054) showed significant efficacy (P = 0.0004, 48% TGI). However, in the HER-2 IHC 2+ EC044 model, targeted therapy with Trastuzumab induced significant tumor regression (93% tumor reduction, *p*<0.0001). Surprisingly, significant anti-tumor activity was not observed with Trastuzumab treatment in a second HER-2 IHC 2+ model (EC039, *p* = 0.229).

### Gene mutation screening in PDECX models

**Figure 4:**
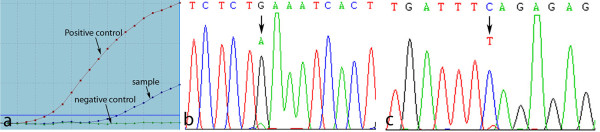
PIK3CA gene mutation in model EC039 and the corresponding primary tumor identified by ARMS method (a) and confirmation of PIK3CA gene mutation in 1624 nucleotide, corresponding to codon 542 (GAA542AAA/E542K) in exon 9 helical domain of PIK3CA gene by PCR-direct sequencing (b: Forward, c: Reverse).

To explore potential Trastuzumab resistance mechanisms in model EC039, we screened for mutations in a panel of genes (EGFR, K-ras, B-raf and PIK3CA) across all 5 PDECX models and their corresponding patient EC tissues. Screened gene locations included; exons 18, 19, 20, 21 of EGFR gene, exons 2 and 3 of K-ras gene, exons 11 and 15 of B-raf gene, and exons 9 and 20 of PI3KCA gene. Interestingly, PIK3CA G1624A (E542K) mutation was identified within exon 9 of the PIK3CA gene helical domain in both EC039 model and the corresponding patient’s EC tissue. This was identified by mutant-enriched liquid chip PCR and ARMS methods (Figure 4a), and subsequently confirmed by direct-sequencing (Figure 4b, c). No other mutations were detected in these 5 PDECX mouse models and the corresponding patient ESCC tissues.

### Western blotting for EC039 and EC044 models

**Figure 5:**
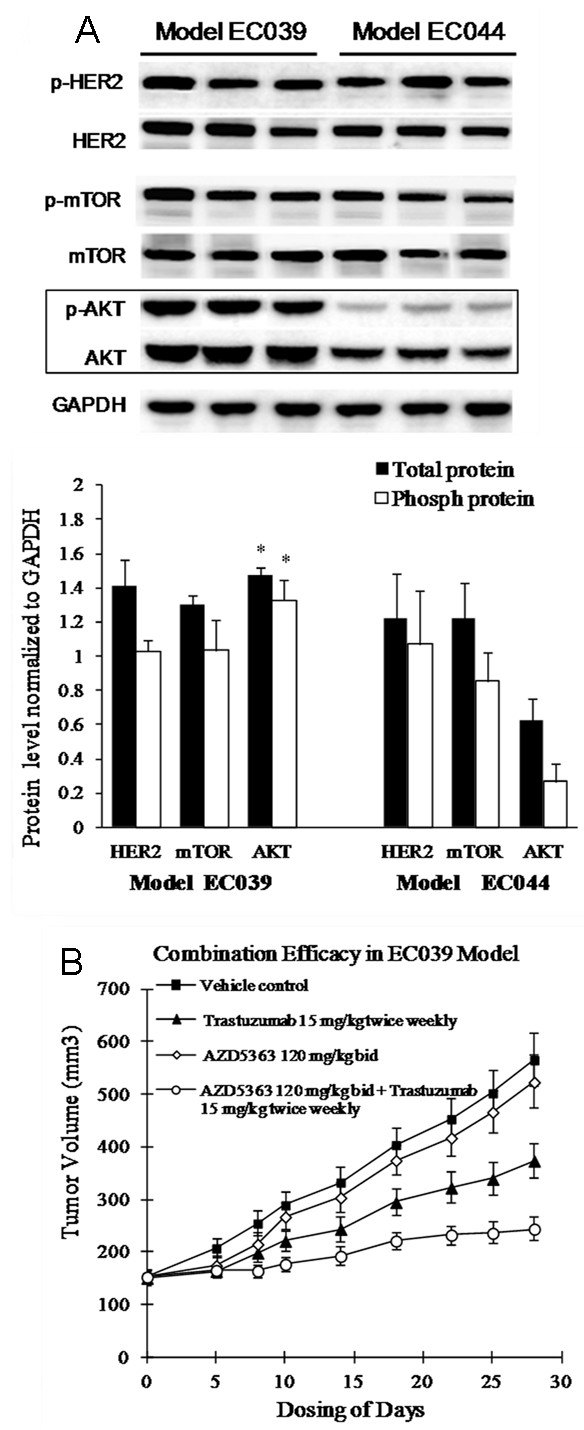
**AKT activation in PIK3CA mutation model and AKT inhibition desensitizes model EC039 to Trastuzumab therapy. A**. Western blot analyses of EC039 and EC044 PDECX models. Three xenograft tumors in both PIK3CA mutation EC039 and wild type EC044 model were harvested, and total proteins were extracted and analyzed with the indicated antibodies. Blots were quantified by density scanning and the graphed data (Mean + SD) represents the quantification of the ratio of protein expression to GAPDH. Significant changes (p < 0.0004) between the two models are labeled as *. **B.** Combination with Trastuzumab and AKT selective inhibitor AZD5363 in PIK3CA mutation model EC039.

Due to observing dramatically different Trastuzumab anti-tumor responses within two xenograft models with the same HER-2 level (2+), but differing PIK3CA mutation status, we sought to determine the AKT pathway activation states within tumor lysates from these models. Western blotting was performed and the quantified data represented in Figure 5A. Significant increases in the protein levels of AKT and pAKT were observed in model EC039 compared to model EC044 (p<0.0004). Our data clearly demonstrate AKT pathway activation as a consequence of PIK3CA mutation in model EC039, compared with the PIK3CA wild type model EC044.

### AKT inhibition resensitises model EC039 to Trastuzumab therapy in vivo

To further test our hypothesis that PIK3CA mutation was responsible for the resistance to Trastuzumab therapy in a HER-2 positive esophageal PDECX model, we performed an anti-tumor efficacy combination study using Trastuzumab and the small molecule AKT inhibitor, AZD5363 [14]. Trastuzumab monotherapy (15 mg/kg twice weekly) resulted in a modest 45% TGI (*P* = 0.003) whilst AZD5363 therapy alone (120 mg/kg bid) gave a non-significant TGI of 10% (*P* = 0.281). The combination of both agents together however, resulted in a significant synergistic anti-tumor effect, generating TGI of 78% (*P* <0.0001) (Figure 5B).

## Discussion

It is well established that patient-derived xenograft mouse models can better represent human tumors through increased diversity of molecular lesions and the preservation of three-dimensional tumor-stromal cell components and interactions [15]. Although some patient-derived xenograft models, e.g., NSCLC [16,17], melanoma [18], colon cancer [19], breast cancer [20], HCC [21,22], GIST [23], have been established and used for evaluation of anti-tumor compounds [24], the availability of patient-derived EC xenograft models is limited. Previous studies have however, developed xenograft EC models by implanting human EC cell lines into mice [25,26] and the first objective of this study was to develop novel PDECX models. After successfully establishing 25 PDECX mouse models through implantation of 54 patient EC samples, 5 PDECX mouse models were selected for evaluation of Trastuzumab anti-tumor efficacy based on HER-2 expression levels. Although the five models displayed varying growth rates (Figure 1), this was not related to differentiation and HER-2 positivity (Table 1). Indeed, the varying growth rates of patient-derived models are likely related (at least in part), to the requirement to directly implant tumor fragments, rather than standardized defined numbers of tumor cells.

Trastuzumab anti-tumor efficacy correlates well with HER-2 expression in patients with breast [6,27-29] and gastric cancer [7,24,30]. Esophageal carcinoma represents another disease with a high frequency of tumor HER-2 expression and gene amplification [8,9,11]. In this setting, the frequency of patients with tumor HER-2 positive expression ranges from 0 to 56% [8,9], whilst the frequency of HER-2 gene amplification varies from 5% to 35% [8,9,11]. In addition, the predominant histological subtype of esophageal carcinoma differs between Eastern and Western countries, and adenocarcinoma has been shown to exhibit higher rates of HER-2 protein expression positivity and gene amplification compared to squamous cell carcinoma [11]. Accordingly, most current preclinical and clinical studies are focused on esophageal adenocarcinoma and literature data supports a correlation between HER-2 gene expression (or amplification) and Trastuzumab efficacy in esophageal adenocarcinoma (preclinical and clinical phase I/II data [10,31] however, such data is rare in esophageal squamous cell carcinoma [32]. In order to address the therapeutic efficacy of Trastuzumab in esophageal squamous cell carcinoma preclinical studies, we characterized 5 PDECX mouse models for HER-2 gene amplification and protein expression. In this study, we did not observe strong positive staining for HER-2 expression (3+) or gene amplification (FISH score ≥ 6) in the 25 established PDECX mouse models. Two PDECX mouse models (EC039 and 044) were positive (IHC 2+) for HER-2 expression and had relatively high *HER-2* copy number (~3.9). *HER-2* gene copy number and protein expression were highly consistent between the 5 PDECX mouse models and their corresponding patient’s EC tissues (Figure 2), suggesting that the PDECX mouse models better represent clinical EC disease, compared to standard cell line-derived xenografts.

Clinically, approximately 20% of esophageal adenocarcinoma patients who were HER-2 IHC 2+ and FISH negative demonstrated a complete clinical response [10]. Mimura *et al.* have also demonstrated that HER-2 expressing esophageal SCC cells could be killed by Trastuzumab-mediated ADCC and that anti-proliferative activity reflected the degree of HER-2 expression detected by flow cytometry, but not by HercepTest nor fluorescence in situ hybridization analysis. To determine if HER-2 positive (IHC 2+) and FISH negative esophageal squamous cell carcinoma could be responsive to Trastuzumab monotherapy, we tested Trastuzumab anti-tumor efficacy in 5 established PDECX mouse models with varying degrees of HER-2 expression and gene copy number. Unsurprisingly, there was no tumor regression, and no or marginal response to Trastuzumab treatment in the HER-2 negative PDECX models. Our data demonstrated that Trastuzumab induced complete tumor regression in one of the HER-2 positive models (IHC 2+) (Figure 3 and Table 1), but generated no significant anti-tumor response in the other. Whilst we believe that this potent anti-tumor effect is a direct consequence of anti-proliferative activity, we note that Trastuzumab has been observed to exert both mild anti-angiogenic and anti-metastatic effects [33] however, we were unable to assess changes in these phenotypes within the studies documented here.

To explore the underlying mechanism behind these contrasting Trastuzumab responses, additional genetic changes were analyzed. It is well known that some key gene mutations including EGFR, K-ras, B-raf, PIK3CA, often affect the efficacy of molecular-targeted therapies [34,35]. In clinical studies of metastatic colon cancer, patients with wild type K-ras, B-raf and PIK3CA genes gain benefit from cetuximab therapy[34], whilst lung cancer patients with mutations in exons 19 and 21 of the EGFR gene benefit from Iressa or Tarceva therapy. Furthermore, mutations in ESCC patients have been recently reported; 16% of ESCC patients (5/30 cases) harbor K-ras gene mutations [36], 14% of ESCC patients (7/50 cases) contain EGFR mutation [37], and 2.2-11.8% of ESCC patients have gene mutations in exon 9 of the PIK3CA gene [38,39]. These findings suggest that gene mutation(s) may play a key role in resistance to Trastuzumab treatment. After screening for EGFR, K-ras, B-raf and PIK3CA gene mutations in the 5 PDECX mouse models and their corresponding primary tumors, we found that model EC039 (and the corresponding patient tissue) had a helical domain mutation (G1624A, E542K) in exon 9 of the PIK3CA gene (Figure 4 and Table 1). This mutation is known to activate downstream signaling of the AKT pathway [40,41]. As such, this result likely explains why the HER-2 positive (IHC 2+) model EC039 was unresponsive to Trastuzumab treatment, whilst model EC044 (HER-2 positive (IHC 2+)) displayed tumor regression. PIK3CA activating mutations have been reported to associate with shorter time to progression (TTP) in Trastuzumab-treated breast cancer [42]. More recently, particular attention has been paid to the role of the PIK3CA gene in Trastuzumab resistance. Mutations in exon 9 and 20 of the PIK3CA gene have been observed in 8% to 40% of breast cancers [43-45]. Furthermore, an association between PIK3CA mutation and resistance to Trastuzumab therapy in HER-2-amplified breast cancer cell lines has also been reported [46]. Berns *et al*. have also suggested that oncogenic mutations of PIK3CA may render breast cancers more resistant to treatment with Trastuzumab [47], and Eichhorn *et al*. have further shown that mutational activation of PIK3CA can similarly render cells more resistant to the recently approved anti-HER2 agent Lapatinib. In this situation however, Lapatinib resistance caused by the dominant activating mutations in PIK3CA can be effectively reversed by treatment with the PI3K/mTOR inhibitor, NVP-BEZ235 [48]. Moreover, the AKT inhibitor AZD5363 increases the efficacy of lapatinib and/or trastuzumab in xenografts derived from HER2+ breast cancer cell lines harboring PIK3CA mutation [14]. In our study, we observed strong AKT pathway activation in the PIK3CA mutation model EC039 (Figure 5A) compared to wild type model EC044, suggesting that activated AKT pathway signaling likely contributes to Trastuzumab resistance in our efficacy study. To our knowledge, we are the first to describe a PIK3CA gene mutation which causes resistance to Trastuzumab therapy in a preclinical PDECX mouse model, and which can be partly resensitised using a combination of Trastuzumab and AKT inhibitor (Figure 5B). The inability to achieve full tumor regression in this combination study may be a consequence of only blocking PI3K pathway signaling downstream through AKT, thus still allowing PDK1, PKC and Rac signaling to occur.

In summary, we have established novel xenograft models derived from patient esophageal squamous cell carcinoma, and have used these clinically relevant animal models to investigate Trastuzumab efficacy in HER-2 expressing ESCC. Our results demonstrate that a non-amplified, HER-2 IHC 2+ model responds completely to Trastuzumab targeted therapy, and importantly, that PIK3CA gene mutation negatively associates with Trastuzumab anti-tumor efficacy in a xenograft model of ESCC. Moreover, resensitization to Trastuzumab therapy is achieved when treatment is combined with an AKT inhibitor to suppress PIK3CA pathway signaling. Taken together, this study illustrates that Trastuzumab can induce regression in a HER-2 positive tumor, and that PIK3CA mutation (in a HER-2 positive background) may be a potential resistance mechanism to Trastuzumab treatment in pre-clinical patient-derived EC xenograft models. We believe that this pre-clinical observation and hypothesis warrants further investigation in a clinical study.

## Competing interests

The authors declare that they have no competing interests.

## Authors’ contributions

WX performed the mouse model studies, ZJ and JQ participated study design and drafted the manuscript. PG revised the manuscript. ZR and XS collected the fresh tissue specimens. LJ and XL performed PDECX model establishment and efficacy studies. ZL and HD participated in immunochemistry work. SX and YX completed the FISH studies, whilst ZG and TY participated in the sequence alignment. LS performed the statistical analysis and HJ checked the results. LY and XC finished the PCR experiments. HY and GD conceived of the study, and participated in its design and coordination and helped to draft the manuscript. All authors read and approved the final manuscript.
